# *Enterococcus faecalis* Clones in Poultry and in Humans with Urinary Tract Infections, Vietnam

**DOI:** 10.3201/eid1807.111754

**Published:** 2012-07

**Authors:** Louise Ladefoged Poulsen, Magne Bisgaard, Nguyen Thai Son, Nguyen Vu Trung, Hoang Manh An, Anders Dalsgaard

**Affiliations:** University of Copenhagen, Copenhagen, Denmark (L.L. Poulsen, M. Bisgaard, A. Dalsgaard);; Military Medical University, Ha Dong, Hanoi, Vietnam (N.T. Son, H.M. An);; and Hanoi Medical University, Hanoi (N.V. Trung)

**Keywords:** Enterococcus faecalis, zoonoses, urinary tract infection, bacteria, humans, poultry, chickens, Vietnam, antimicrobial resistance, UTI

## Abstract

*Enterococcus* spp. as pathogens have increased, but the sources of infection often remain unclear. To investigate whether poultry might be a reservoir for *E. faecalis*–associated urinary tract infections (UTIs) in humans, we characterized *E. faecalis* isolates from patients in Vietnam with UTIs during January 2008–January 2010 and poultry living in close contact with them by multilocus sequence typing (MLST), pulsed-field gel electrophoresis, analysis of antimicrobial drug susceptibility patterns, and sequencing of virulence genes. In 7 (23%) of 31 UTI cases, we detected identical MLST, indistinguishable or closely related pulsed-field gel electrophoresis patterns, and similar antimicrobial drug susceptibility patterns. Isolates from urine and poultry showed identical virulence gene profiles, except for 1 variation, and individual genes showed identical sequences. The homology of isolates from urine and poultry further indicates the zoonotic potential and global spread of *E. faecalis* sequence type 16, which recently was reported in humans with endocarditis and in pigs in Denmark.

## Abstract 2

Transmission routes and reservoirs need to be elucidated.

Enterococci are commensals of the human and animal gastrointestinal tract and opportunistic pathogens that cause urinary tract infections (UTIs), endocarditis, and sepsis (*1*). Nosocomial infections caused by enterococci have increased; these pathogens are now the third most common at hospitals after *Escherichia coli* and *Staphylococcus aureus* (*2*); and enterococci are frequently recorded as the cause of UTIs, wound infections, bacteremia, and endocarditis (*3**–**6*).

The sources of enterococcal infections in humans are not clear, but animal reservoirs have been suggested (*2**,**4**,**7**–**9*). A study comparing enterococcal isolates from 4 European countries and the United States demonstrated that *E. faecalis* isolated from pigs in Portugal had pulsed-field gel electrophoresis (PFGE) patterns identical to those of multidrug-resistant isolates at hospitals in Spain, Italy, and Portugal, all of which were shown by multilocus sequence typing (MLST) to belong to sequence type (ST) 6 (*7*). In Denmark, high-level gentamicin-resistant *E. faecalis* of ST16 with an identical PFGE pattern was isolated from pigs and from humans with endocarditis (*9*). Identical and closely related PFGE patterns were demonstrated by isolates from humans and from pork and chicken meat in the United States, all of which contained high-level gentamicin-resistant genes (*4*). Our objective was to characterize epidemiologically related *E. faecalis* isolated from humans with UTIs and from poultry living in the same households in Vietnam to evaluate the zoonotic potential of *E. faecalis*.

## Materials and Methods

### Recruitment of Patients, Urine Collection, and Bacterial Culture of Urine

Urine samples were collected during January 2008–January 2010 at the Military Medical University, Hospital 103, in Ha Dong, Hanoi. Patients with clinical symptoms of UTI (i.e., >1 of the following symptoms: frequent urination; painful urination; hematuria; cloudy urine; or pain in pelvic area, flank, or low back) were referred from nearby pharmacies and informed about the project. A midstream urine sample was collected at the hospital under supervision of a nurse. Only patients with uncomplicated UTIs were included; patients reporting underlying diseases, such as hematologic disorders, respiratory infections, diarrhea, diabetes, cancer, HIV/AIDS, liver cirrhosis, alcoholism, anatomic malformations of urinary tract, nephrolithiasis, or urolithiasis were excluded, as were patients with hospital-acquired UTIs. The urine was cultured immediately after collection. Thirty-one UTI patients met the study criteria of having *E*. *faecalis* CFU >10^3^/mL isolated from a urine sample in pure culture and were raising poultry in their households.

The urine samples were cultured on Flexicult agar plates (Statens Serum Institut, Copenhagen, Denmark), where *E. faecalis* grows as small green/blue-green colonies and *E. faecium* as small green colonies (*10*). Three colonies were isolated from each UTI patient. All 31 participants were interviewed when urine samples were collected. Personal information recorded included age, sex, and underlying diseases. The following clinical symptoms were recorded: frequent urination, painful urination, cloudy urine, blood in urine, pain in pelvic area, flank pain, pain in low back, and fever. In addition, information about duration of symptoms; previous UTIs; and medical treatment before arrival at the hospital, including type of antimicrobial drug used, was recorded.

Species identification of all 31 presumptive *E. faecalis* isolates from urine and 83 isolates from poultry were confirmed by species-specific PCR as described by Dutka-Malen et al. (*11*). Only isolates identified as *E. faecalis* by PCR were further characterized.

All study participants were informed orally and in writing about the study and provided written consent. The ethics committee at Army Hospital 103 approved the study protocols.

### Collection of Cloacal Swabs from Poultry

When a urine sample was positive for *E. faecalis*, the patient’s household was visited within 1 week, and cloacal swabs were taken from 2–4 chickens in the household. Fecal samples were taken with a sterile cotton swab and immediately placed in Cary-Blair media (Oxoid, Basingstoke, Hampshire, UK) for transportation to the laboratory. Samples were then streaked on Slanetz and Bartley agar medium (Merck, Darmstadt, Germany) the same day and incubated for 24–48 h at 37°C. Subsequently, 2 individual colonies were randomly selected and subcultured on nonselective LB-agar, Lennox plates (Difco, Becton Dickinson, Sparks, MD, USA), which were incubated overnight at 37°C to obtain pure cultures. Colonies were then grown in brain–heart infusion broth (Oxoid) overnight at 37°C and stored for further characterization at –80°C in cryotubes containing 30% glycerol.

### MLST and PFGE

To investigate whether isolates of *E. faecalis* from urine and poultry belonged to identical STs, we characterized isolates from urine and poultry by MLST. Urine isolates were characterized by sequencing of all 7 housekeeping genes used in the MLST scheme: *gdh*, *gyd*, *pstS*, *gki*, *aroE*, *xpt*, and *yqil*. To confirm that the UTIs were caused by a single strain, 1 additional colony from 9 (29%) of 31 urine samples was characterized by sequencing the *gki* and *yqil* genes. Two isolates from each chicken were characterized by sequencing the *gki* and *yqil* genes. When sequences of both genes in 2 isolates corresponded to the sequence of the same genes in the urine isolate, which occurred in 11 cases, 1 of the 2 isolates from poultry was randomly selected and further characterized. When gene sequences in only 1 isolate from poultry were identical to the isolate from urine, the isolate was further characterized. Primers and PCR conditions are described on the *E. faecalis* MLST website (http://efaecalis.mlst.net/). Amplicons were sequenced in both directions by Macrogen (Seoul, South Korea). DNA sequences obtained were assembled using CLC Main Workbench 5.2 software (CLC bio, Aarhus, Denmark) and compared with published alleles, and an ST was assigned to each strain (http://efaecalis.mlst.net/). PFGE was performed as described (*12*) by using the restriction enzyme *sma*I (New England BioLabs, Ipswich, MA, USA).

### Virulence Genes

The presence and sequence of the following 6 virulence genes were used to further characterize the isolates from urine and poultry: *asa*1, *CylA*, *efaA*, *Esp*, *gelE*, and EF0591 (*13*). After detecting the virulence genes by PCR (*13*), we sequenced the genes in both directions using Macrogen. DNA sequences were compared, and possible nucleotide differences were calculated by using Smith-Waterman local alignment (EMBOSS) available online from the European Bioinformatics Institute: (www.ebi.ac.uk/).

### Antimicrobial Drug Susceptibility Testing

MICs were determined for 16 antimicrobial drugs for comparison analyses by using the Sensititer system (Trek Diagnostics Systems, East Grindstead, UK) according to the manufacturer’s guidelines. These drugs were ampicillin (2–32 μg/mL), avilamycin (4–32 μg/mL), chloramphenicol (2–64 μg/mL), daptomycin (0.25–16 μg/mL), erythromycin (0.5–32 μg/mL), gentamicin (16–1,024 μg/mL), kanamicin (128–2,048 μg/mL), linezolid (0.5–8 μg/mL), moxifloxacin (0.25–8 μg/mL), penicillin (2–32 μg/mL), salinomycin (2–16 μg/mL), streptomycin (64–2,048 μg/mL), quinupristin-dalfopristin (0.25–16 μg/mL), tetracycline (1–32 μg/mL), tigecycline (0.015–2 μg/mL), and vancomycin (1–32 μg/mL).

## Results

In 7 (23%) of 31 UTI cases, *E. faecalis* isolated from patient urine and poultry demonstrated identical STs and an indistinguishable (4 pairs) or closely related PFGE pattern (3 pairs, defined as showing <3 fragment difference) (Figure). In addition, antimicrobial drug susceptibility patterns were similar, and only 1 variation was found in the virulence gene profiles (Table 1, Table 2). Five of these 7 patients reportedly had a profession where they worked with poultry. A total of 22 patients who did not share a clone of *E. faecalis* found in poultry in their household reported working with poultry.

**Figure:**
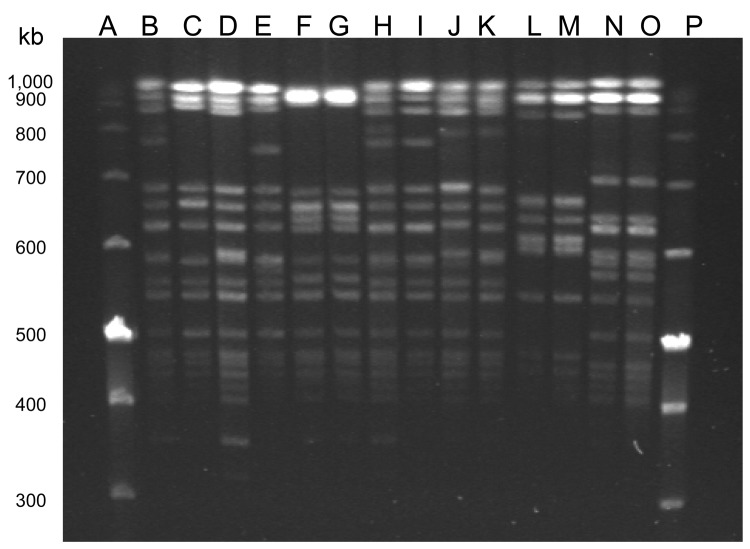
Pulsed-field gel electrophoresis of *Sma*I-digested *Enterococcus faecalis* isolated from humans with urinary tract infections and from poultry in the same houselhold, Vietnam, January 2008–January 2010. Lanes A and P are molecular weight markers. Lane B, isolate 90U; lane C, isolate 90P; lane D, 122U; lane E, 122P; lane F, 186U; lane G, 186P; lane H, 191U; lane I, 191P; lane J, 204U; lane K, 204P; lane L, 217U; lane M, 217P; lane N, 221U; and lane O, 221P.

**Table 1:** MLST, PFGE, and virulence gene profiles for *Enterococcus faecalis* isolated from humans with urinary tract infections and poultry from the same households, Vietnam, January 2008–January 2010*

Strain†	Source	MLST type	PFGE pattern	Virulence genes						Duration of symptoms, mo
				*asa1*	*CylA*	*efaA*	*Esp*	*gelE*	EF0591	
90U	Urine	16	A1	–	+	+	+	–	+	1
90P	Poultry	16	A2	+	+	+	+	–	+	NA
122U	Urine	16	A2	+	+	+	+	–	+	7
122P	Poultry	16	A1	+	+	+	+	–	+	NA
186U	Urine	93	A3	–	–	+	–	–	–	24
186P	Poultry	93	A3	–	–	+	–	–	–	NA
191U	Urine	413	A3	–	+	+	+	–	+	24
191P	Poultry	413	A1	–	+	+	+	–	+	NA
204U	Urine	16	A1	–	+	+	+	–	+	2
204P	Poultry	16	A1	–	+	+	+	–	+	NA
217U	Urine	415	A3	+	–	+	–	+	–	0.5
217P	Poultry	415	A3	+	–	+	–	+	–	NA
221U	Urine	141	A6	–	–	+	–	+	–	120
221P	Poultry	141	A6	–	–	+	–	+	–	NA

*MLST, multilocus sequence typing; PFGE, pulsed-field gel electrophoresis; +, positive; –, negative; NA, not applicable. †All isolates except 221U were recovered from female patients. Strains isolated from patients with urinary tract infections are designated as U and strains isolated from poultry as P.

**Table 2:** Antimicrobial drug susceptibility (MIC) testing of *Enterococcus faecalis* isolated from humans with urinary tract infections and poultry in the same household, Vietnam, January 2008–January 2010*

Strain	Antimicrobial drug and test interval, µg/mL								
	CHL, 2–64	DAP, 0.25–16	ERY, 0.5–32	GEN, 16–1,024	KAN, 128–2,048	MXF, 0.25–8	STR, 64–2048	Q-D, 0.25–16	TET, 1–32
90U	64	4†	>32	>1,024	>2,048	<0.25	>2,048†	16	>32
90P	>64	>16†	32	512	>2,048	<0.25	128†	16	>32
122U	64	4	>32	64†	>2,048	<0.25	128†	16	>32
122P	>64	4	>32	1,024†	>2,048	<0.25	>2,048†	16	>32
186U	4	4	>32†	<16	<128	<0.25	<64	16	>32†
186P	4	8	<0.5†	<16	<128	<0.25	<64	16	<1†
191U	32	4	>32	>1,024	>2,048	<0.25	>2,048	16	32†
191P	64	8	>32	512	>2,048	<0.25	>2,048	16	<1†
204U	64†	4	>32†	>1,024†	>2,048†	<0.25†	>2,048†	16†	32
204P	4†	8	4†	<16†	<128†	2†	<64†	0.5†	32
217U	4	4	<0.5	32	<128	<0.25	256	8	<1
217P	4	4	<0.5	<16	<128	<0.25	256	8	<1
221U	64	4	<0.5	<16†	<128	<0.25	128	8	>32
221P	64	4	1	64†	<128	<0.25	128	8	>32

*CHL, chloramphenicol; DAP, daptomycin; ERY, erythromycin; GEN, gentamicin; KAN, kanamicin; MXF, moxifloxacin; STR, streptomycin; Q-D, quinupristin-dalfopristin; TET, tetracycline. †>1 dilution difference between urine and poultry strain.

### MLST

Sequencing the 7 housekeeping genes in the 31 *E. faecalis* strains showed the following 14 STs: 4, 16, 17, 93, 116, 136, 141, 314, 410, 411, 412, 413, 415, and 417, with ST16 shown by 16 (51.6%) isolates. Three isolates belonged to ST4, and each of the remaining STs was represented by only 1 isolate. In 7 of 31 households, the same ST was obtained from poultry and urine (Table 1). In 3 households, ST16 was isolated from urine and poultry. In the remaining 4 households, STs 93, 141, 413, and 415 were identified (Table 1). Because each pair of isolates from all selected patients (28%) showed identical *gki* and *yqil* gene sequences, we concluded that the UTI cases were associated with 1 *E. faecalis* strain.

### PFGE

We detected 6 PFGE patterns (A1–A6). Of these, 4 pairs from urine and poultry from the same households showed indistinguishable patterns (Table 1; Figure).

### Antimicrobial Drug Susceptibility Testing

When we compared isolates from urine and poultry from individual households, we detected similar MICs of each tested antimicrobial drug, showing a 1-dilution factor deviation (Table 2). For several isolates, an MIC could not be established because the MIC fell outside the test intervals. We detected different MICs for 7 antimicrobial drugs when we compared strains 204U and 204P. All isolates were fully susceptible (lowest or second lowest MIC tested) to ampicillin, avilamycin, linezolid, penicillin, salinomycin, tigecycline, and vancomycin (results not shown in Table 2).

### Virulence Genes

PCR for the 6 virulence genes showed that the isolates from urine and poultry from an individual household contained identical virulence genes that varied from 1 to 5 genes, except for 1 household in which the isolate from urine (90U) did not contain the *asa*1 gene (Table 1). When we compared the DNA sequences from the epidemiologically related urine and poultry strains, we found that all 23 sequenced gene pairs showed 100% similarity.

## Discussion

We document isolation of the same clone of *E. faecalis* in urine and poultry from the same households in which patients had close contact with the poultry. The potential for zoonotic transmission of *E. faecalis* has been suggested, but to our knowledge, only epidemiologically unrelated isolates have been investigated (*3**,**4**,**7**–**9**,**14*).

Most of the isolates in our study belonged to ST16, which has been isolated from animals and humans, including clinical and nonclinical isolates (*14*). ST93 was isolated from a patient with an ulcer in Poland and from an unknown source in the United States, and ST141 was isolated from chickens in Denmark and from a blood sample of a hospitalized person in Poland (http://efaecalis.mlst.net/).

When we interpreted PFGE patterns for their relatedness using criteria suggested by Tenover et al. (*15*), we found 4 pairs of *E. faecalis* strains with indistinguishable band patterns that could be “considered to represent the same strain” (*15*). From 3 individual households, isolates from urine and poultry showed PFGE patterns with 1 or 2 band differences and thus can be considered closely related (*15*). These identical or closely related PFGE patterns, together with the supporting findings by MLST and virulence gene profiling, suggest that *E. faecalis* might be transmitted from poultry to humans, causing UTIs. However, the finding of similar isolates from humans and poultry also could result from sharing a common clone of *E. faecalis*. ST16 has been reported from various epidemiologically unrelated human and animal sources (*14*), which could indicate a common clone in humans and animals. Because no data about ST16 in the environment are available, an environmental reservoir cannot be ruled out.

Because 27 of the 31 patients reported having contact with poultry through their work, contact with poultry outside the household environment cannot be excluded as the source of *E. faecalis*. Epidemiologic risk factor studies are needed to document actual transmission routes.

The variation found in resistance patterns might have resulted from exposure to different antimicrobial drugs, resulting in different selection pressure on *E. faecalis* in the human and poultry hosts. The 7 patients studied had UTI symptoms for an average of 514 days (range 5 days–10 years), which is unusually long for UTI (Table 1). Although self-medication is well established to be a common practice in Vietnam (*16*), only 2 of the 7 patients acknowledged use of antimicrobial drugs to treat their UTI symptoms before they participated in the study (data not shown). Over time, patients tend to forget what kind of medication they received. Furthermore, the questionnaire asked only whether antimicrobial drugs were used against UTI, not whether they were used to treat other diseases. In addition, poultry might have been exposed to antimicrobial drugs through growth promoters added in the feedstuff and during therapeutic or preventive treatments, but information about such use was not available.

In most Western countries, contact with poultry occurs mainly through handling and consumption of poultry meat. However, the risk for zoonotic transmission of *E. faecalis* from poultry meat remains to be investigated. Thus, similar studies and risk factor studies should be conducted in more countries to evaluate the effect on zoonotic transmission of differences in human habits of poultry consumption and contact with poultry. In addition, animals other than pigs and poultry should be investigated as sources of zoonotic *E. faecalis* transmission. Finally, we cannot exclude the possibility that *E. faecalis* pathotypes found in poultry might represent transmission from humans, e.g., in this study, from UTI patients. However, poultry as carriers of ST16 has been documented (*17*), and it seems more likely that humans are exposed to poultry litter than that poultry are exposed to human feces.

We did not investigate the route of *E. faecalis* transmission, but the route could be colonization of the human intestine and subsequently ascending the urethra as reported for *E. coli* (*18*). Further studies are required to explain routes of transmission. The emergence of enterococci as causes of human infections and their resistance to some of the crucial antimicrobial drugs used for human treatment emphasizes the need to elucidate transmission routes and reservoirs for the enterococci and their resistance genes (*5**,**6**,**19**–**21*).
